# Brain Activation for Audiovisual Information in People With One Eye Compared to Binocular and Eye-Patched Viewing Controls

**DOI:** 10.3389/fnins.2020.00529

**Published:** 2020-05-21

**Authors:** Stefania S. Moro, Diana J. Gorbet, Jennifer K. E. Steeves

**Affiliations:** ^1^Department of Psychology, York University, Toronto, ON, Canada; ^2^Centre for Vision Research, York University, Toronto, ON, Canada; ^3^The Hospital for Sick Children, Toronto, ON, Canada; ^4^School of Kinesiology and Health Science, York University, Toronto, ON, Canada

**Keywords:** monocular enucleation, audiovisual processing, multisensory, sensory deprivation, fMRI

## Abstract

Blindness caused by early vision loss results in complete visual deprivation and subsequent changes in the use of the remaining intact senses. We have also observed adaptive plasticity in the case of partial visual deprivation. The removal of one eye, through unilateral eye enucleation, results in partial visual deprivation and is a unique model for examining the consequences of the loss of binocularity. Partial deprivation of the visual system from the loss of one eye early in life results in behavioral and structural changes in the remaining senses, namely auditory and audiovisual systems. In the current study we use functional neuroimaging data to relate function and behavior of the audiovisual system in this rare patient group compared to controls viewing binocularly or with one eye patched. In Experiment 1, a whole brain analysis compared common regions of cortical activation between groups, for auditory, visual and audiovisual stimuli. People with one eye demonstrated a trend for increased activation for low-level audiovisual stimuli compared to patched viewing controls but did not differ from binocular viewing controls. In Experiment 2, a region of interest (ROI) analysis for auditory, visual, audiovisual and illusory McGurk stimuli revealed that people with one eye had an increased trend for left hemisphere audiovisual activation for McGurk stimuli compared to binocular viewing controls. This aligns with current behavioral analysis and previous research showing reduced McGurk Effect in people with one eye. Furthermore, there is no evidence of a correlation between behavioral performance on the McGurk Effect task and functional activation. Together with previous behavioral work, these functional data contribute to the broader understanding of cross-sensory effects of early sensory deprivation from eye enucleation. Overall, these results contribute to a better understanding of the sensory deficits experienced by people with one eye, as well as, the relationship between behavior, structure and function in order to better predict the outcome of early partial visual deafferentation.

## Introduction

Complete visual deprivation from blindness leads to adaptive changes in other sensory systems. For example, congenitally blind individuals have shorter response times for auditory discrimination tasks (Röder et al., 1999), faster processing of language (Röder et al., 2002), enhanced sound localization (Lessard et al., 1998) and enhanced tactile perception (Sathian, 2000; Goldreich and Kanics, 2003) compared to sighted individuals. These adaptations suggest that underlying physiological changes have occurred within sensory systems to support such behavioral enhancements. It is possible that visual cortex is recruited or reorganized by other sensory systems in the congenitally blind. Neuroimaging studies have shown activation of visual cortex for sensory stimuli normally processed elsewhere in the brain such as audition (Collignon et al., 2009; Merabet et al., 2009), sound localization (Weeks et al., 2000), and tactile perception and Braille reading (Sadato et al., 1996; Cohen et al., 1997; Buchel et al., 1998; Kupers et al., 2007). Not all recruitment or reorganization results in adaptive change as evidence for the disruption of complementary senses when the visual system is compromised also exists. For example, some have shown congenitally blind individuals have decreased sound localization accuracy in the vertical plane (Lewald, 2002), and horizontal plane (Gori et al., 2014) or decreased distance judgment of auditory stimuli (Wanet and Veraart, 1985). Overall, it appears that in the case of complete sensory deprivation, specifically blindness, it is possible for other intact sensory systems to be altered and in some cases, to adaptively compensate for the loss of vision. One might ask whether such neuroplasticity also holds true in cases of partial sensory deprivation, such as the loss of one eye early in life?

Partial visual deprivation from unilateral eye enucleation, the surgical removal of one eye, is a unique model for examining the consequences of the loss of binocularity (see Steeves et al., 2008, for a review). It is unlike other forms of monocular visual deprivation such as cataract or strabismus which leave abnormal binocular input and contributes to competitive binocular interactions. Surgically removing the eye completely eliminates all forms of visual input to the brain from that eye leaving a single stream of information to the visual system and a complete lack of competitive binocular interactions (Steeves et al., 2008). Early monocular enucleation is a useful model of study since the visual system may not have been exposed to abnormal visual input from the removed eye.

Monocular enucleation during postnatal visual system maturation leads to both enhancements and reductions in visual function. These differences in outcome appear to align with whether one is measuring visual spatial ability or visual motion processing and oculomotor systems (reviewed in Steeves et al., 2008; Kelly et al., 2013). Visual spatial ability is largely intact while visual motion processing and oculomotor systems show small deficits. More recently, a number of behavioral studies of people who have only one eye have assessed abilities outside of the visual system, specifically, audiovisual abilities. These studies aimed to investigate what types of accommodations the brain might make across the senses after losing half of its visual input (see Steeves et al., 2008; Kelly et al., 2013).

Within the auditory domain, people with one eye have enhanced sound localization in all locations (within 78 degrees to the left or right of straight ahead) except for the extreme periphery compared to control participants who were binocular viewing, eye-patched or had both eyes closed (Hoover et al., 2012). In terms of audiovisual processing, people with one eye do not show the typical pattern of visual dominance when asked to categorize rapidly presented audiovisual targets, but rather, they show equivalent auditory and visual processing suggesting an enhanced relative weighting to the auditory component of bimodal stimuli (Moro and Steeves, 2012). These results persist even when the temporal load is increased in the same task by asking participants to detect and discriminate auditory, visual, or bimodal repetitions in a one-back task (Moro and Steeves, 2013). People with one eye do not differ in the width of their temporal binding window compared to binocular and eye-patched viewing controls, however, they have longer response latencies relative to controls indicating a longer processing time required for this task. Eye-patched controls’ response latencies were intermediate to the two other groups (Moro and Steeves, 2018c). Despite no difference in width of temporal binding window, people with one eye are also less susceptible to the double flash illusion compared to both binocular and eye-patched viewing controls. Furthermore, in that task, people with one eye responded as quickly as binocular and eye-patched viewing controls (Moro and Steeves, 2018c).

People with one eye show no difference in variance of audiovisual localization along the horizontal plane compared to binocular and patched viewing control groups (Moro et al., 2014). However, unlike binocular and eye-patched controls, they take longer to localize unimodal visual stimuli compared to unimodal auditory stimuli (Moro et al., 2014). In terms of audiovisual motion in depth, people with one eye demonstrate the same rate of dynamic visual capture (perception of the direction of an auditory signal to be moving in the direction of the incongruent visual signal despite being asked to respond to the auditory signal alone) (Moro and Steeves, 2018b). Unlike static audiovisual localization, people with one eye have no difference in reaction time or accuracy compared to both control groups for this task (Moro and Steeves, 2018b). Together these audiovisual behavioral studies indicate that perhaps task requirements affect behavioral outcomes for this patient group. Both localization studies used low-level flash and beep stimuli, and people with one eye did not differ in overall performance compared to control groups but did perform slower on tasks with less ecological validity (sounds and images moving along the horizontal plane) (Moro et al., 2014) compared to those moving in depth (Moro and Steeves, 2018b).

To increase ecological validity of audiovisual stimuli, face and voice processing has been studied. People with one eye have increased sensitivity to voices on their own (but not non-human sounds, specifically car horns) in a face-voice and car-horn recognition task (Moro and Steeves, 2019). Perhaps this increased sensitivity to voices compensates for the mild face processing deficits in discriminating feature spacing, the face composite effect and face processing time that have previously been found (Kelly et al., 2012). Finally, people with one eye perceive the illusory McGurk effect less often than binocular viewing controls (Moro and Steeves, 2018a). Additionally, they have no difference in reaction time compared to both control groups (Moro and Steeves, 2018a). Overall, these behavioral results might suggest forms of behavioral adaptation following the reduction of visual input from one eye early in life.

No other lab has investigated changes in brain structure in people who have had one eye removed early in life. Not surprisingly, significant degeneration of the anterior visual system, including decreased optic chiasm volume and width is found in people with one eye compared to binocular controls (Kelly et al., 2014). People with one eye also have an overall decrease in lateral geniculate nucleus (LGN) volume compared to binocular controls as expected but surprisingly, the LGN volume contralateral to the remaining eye is less reduced likely from recruitment of deafferented LGN cells by the intact eye (Kelly et al., 2014). These findings indicate that subcortical level reorganization of the visual system occurs after losing one eye early in life (Kelly et al., 2014). At a cortical level, a subsequent study revealed that, compared to binocular viewing controls, people with one eye have increased surface area and gyrification in visual, auditory and multisensory cortices (Kelly et al., 2015). White matter tracts in the visual and auditory systems of people with one eye were examined using Diffusion tensor imaging (DTI) (Wong et al., 2018, 2019). White matter tracts are greater contralateral to the surgically removed eye in the optic radiations, V1-LGN projections and interhemispheric V1 projections of people with one eye compared to binocular viewing controls, likely a reflection of the differences observed in the LGN volume and optic tract contralateral to the removed eye (Kelly et al., 2014; Wong et al., 2018). Auditory wiring appears more substantial than in controls and, further, the connections between the visual and auditory systems are more intact than expected (Wong et al., 2019). Unlike controls, people with one eye have an asymmetric medial geniculate body (MGB) volume with a larger left than right MGB, regardless of which eye was enucleated perhaps reflecting dominance of left hemisphere in auditory processing (Moro et al., 2015). Taken together, there is moderate cortical, subcortical and wiring alterations of auditory and visual processing following early eye enucleation.

In terms of brain function more recently, our lab found reduced functional activation in people with one eye compared to binocular viewing controls in face-preferential brain regions [left fusiform face area (FFA) and bilateral occipital face area (OFA)] (Kelly et al., 2019). These results complement the mild behavioral face deficits in people with one eye (Kelly et al., 2012). The current study examines audiovisual functional activation in people with one eye compared to binocular and patched viewing controls in two separate experiments. Experiment 1 investigates differences in activation intensity in regions of interest localized by conjunction analysis between groups for low-level audiovisual stimuli and high-level face and voice stimuli. Experiment 2 probes audiovisual regions of interest (ROIs) during the presentation of auditory, visual, audiovisual and illusory McGurk stimuli. We predict that functional activation will reflect our previous behavioral findings, specifically relevant is the absence of a McGurk effect in this group (Moro and Steeves, 2018a). Results from this study will provide a better understanding of how people with one eye process auditory and visual information contributing to better clinical outcomes through cross-sensory accommodations and the promotion of long-term visual health in the remaining eye.

## Materials and Methods

### Participants

#### Monocular Enucleation Group

Seven adult participants who had undergone monocular enucleation (ME) at The Hospital for Sick Children participated in this study (mean age = 34 years, SD = 13 years). All ME participants with one eye had been unilaterally eye enucleated (four right eye removed) due to retinoblastoma, a rare childhood cancer of the retina. Age at enucleation ranged from 4 to 60 months (mean age at enucleation = 23 months, SD = 18 months).

#### Binocular Viewing Control Group (BV)

Ten binocularly intact controls with a mean age of 32 years (SD = 13 years) were tested viewing stimuli out of both eyes.

#### Monocular Viewing Control Group (MV)

Ten binocularly intact participants, with a mean age of 31 years (SD = 16 years), completed the experiments with one eye patched. Participants’ non-preferred eye was patched with a semi-opaque eye covering and translucent tape (five right-eye covered).

All participants (ME, BV, MV) reported normal hearing and normal or corrected-to-normal acuity as assessed by an EDTRS eye chart (Precision Vision^TM^, La Salle, IL, United States) and wore optical correction if needed. All participants gave informed consent prior to their inclusion in the study, which was approved by York University’s Office of Research Ethics.

### Stimuli

#### Audiovisual Blocked Design

##### Low-level stimuli

**FIGURE 1:**
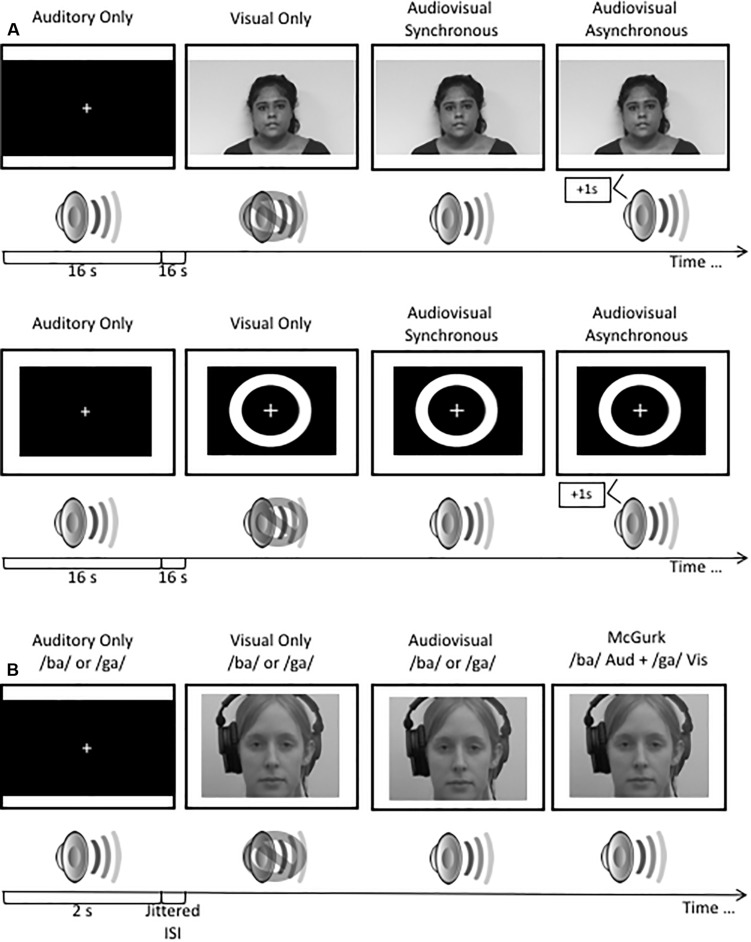
**(A)** A schematic illustration of the stimuli used in the audiovisual blocked design study. **(B)** A schematic illustration of the presentation of stimuli used in the rapid event related design study. Stimuli used in the rapid event related design study have been previously published (see Quinto et al., 2010; Stevenson et al., 2012; Moro and Steeves, 2018a). All visual stimuli were presented to participants in color.

Visual stimuli consisted of a white ring on a black background with a visual fixation cross in the center. Each visual stimulus was 17 ms in length. The auditory stimulus consisted of a 3500 Hz pure tone with a duration of 13 ms. Stimuli were repeated eight times per trial for a duration of 2 s. Trials were presented in a block design. Each block consisted of eight trials (16 s/block). Blocks consisting of visual only, auditory only and audiovisual and asynchronous audiovisual (sound was displaced by 500 ms) stimuli were presented (Figure 1A). A 16 s interstimulus interval of silence and a blank screen was presented between each block.

##### High-level stimuli

Visual stimuli consisted of a 16 s video of a female speaker reading an excerpt from a children’s story. Auditory stimuli consisted of a 16 s audio clip of the female speaker from the video saying the story aloud in a clear and articulate voice. Audiovisual stimuli consisted of two 16 s videos of the female speaker reading the excerpt with the auditory component of the video either synchronously or asynchronously (auditory presented 500 ms after the visual stimulus) paired with the corresponding video. Participants viewed one repetition per trial. Trials were presented in a block design. Each block consisted of one trial (16 s; Figure 1A). A 16 s interstimulus interval consisting of silence and a blank screen was presented after each block.

#### Rapid-Event Related Design

Visual stimuli consisted of two 2 s videos of a female speaker mouthing the syllables “ba” and “ga,” with each presentation containing the entire articulation of the syllable (Quinto et al., 2010; Stevenson et al., 2012; Moro and Steeves, 2018a). Auditory stimuli consisted of 2 s audio clips of the female speaker from the videos saying the syllables “ba” and “ga”. Audiovisual stimuli consisted of two 2 s videos of the female speaker saying the syllables “ba” and “ga,” paired with the corresponding video, respectively. McGurk illusory stimuli consisted of video footage of the female speaker mouthing the “ga” syllable but paired with the auditory sound clip of the female speaker saying “ba” (Figure 1B). Stimuli were presented using a rapid-event related design with jittered interstimulus intervals of variable lengths up to 15 s in order to improve the sampling of the hemodynamic response function (HRF). All stimuli were counterbalanced using OptSeq2 (Greve, 2002). Each stimulus condition was presented 15 times per run.

### Procedure

All participants were scanned at York University’s Sherman Health Science Research Center with a Siemens MAGNETOM Trio 3T MRI scanner (Siemens, Erlangen, Germany) using a 32-channel high-resolution brain array coil. BOLD (blood-oxygen-level dependent) fMRI imaging was utilized to acquire functional images. An echoplanar imaging sequence with the following specifications was used to obtain functional volumes: 35 contiguous axial slices; in-plane resolution 3 × 3 mm; slice thickness 3.5 mm; TR = 2000 ms; TE = 30 ms; imaging matrix 96 x 96; flip angle 90°; FoV = 192 mm. Following the functional scans, a high-resolution whole brain structural image was obtained with a T1 magnetization-prepared rapid gradient echo imaging sequence. The anatomical imaging had the following parameters: 192 slices; in-plane resolution 1 × 1 mm; slice thickness 1 mm; TR 1900 ms; TE 2500 ms; imaging matrix 256 × 256; flip angle 9°; FoV = 256 mm.

Experimental stimuli were presented using VPixx visual testing software (VPixx Technologies Inc., Montréal, QC, United States) via a 33 × 19.5 cm screen inside of the scanner and noise-canceling headphones (MR Confon GmbH, Magdeburg, Germany). Prior to scanning, sound samples were presented to participants to ensure that the sound pressure level was audible and comfortable. Participants were instructed to press a button on a Current Designs 8-Button Bimanual Curved Lines button box (Current Designs, Philadelphia, PA, United States) whenever they perceived the stimuli to be asynchronous to ensure that they were alert and paying attention throughout the task. Each participant performed in seven experimental runs. Five runs consisted of the audiovisual blocked design stimuli and two runs consisted of the rapid-event related McGurk stimuli. All runs were presented to each participant in counterbalanced order.

### Data Analysis

#### Experiment 1: Audiovisual Block Design Whole Brain and ROI Analyses

During MRI data collection, estimates of head motion (translation and rotation) were viewed in real time in the MRI control room to verify that head movement did not exceed 1 mm in any direction. Participants who exceeded this threshold for head motion repeated imaging runs when necessary. For Experiment 1, analysis of MRI data was performed using BrainVoyager v20.6 (Brain Innovation, Maastricht, Netherlands). Preprocessing of the audiovisual block design functional data included slice time correction followed by motion correction, and linear trend removal. Motion correction used a trilinear/sinc interpolation method with the first functional volume used as the reference. Plots of head motion estimates and movies of head motion over the course of each functional run were generated and visually inspected to confirm that all experimental runs were free from head movements over 1 mm in any direction and free from obvious hardware-related artifacts. Spatial smoothing was applied to each functional run using a 6 mm full width half maximum isotropic kernel. Functional runs were coregistered with corresponding high resolution T1-weighted anatomical images. Images were transformed from subject-space to Talairach template space.

Design matrices for use in general linear model (GLM) analyses were constructed for each participant using a boxcar design convolved with a hemodynamic response function. Within the design matrices, stimulation timing protocols were used to define separate predictors for each of the eight experimental conditions (i.e., low-level and high-level auditory, visual, synchronous audiovisual, and asynchronous audiovisual stimuli). Non-parametric permutation testing was performed for whole brain group comparisons of each condition using the randomize plug-in for BrainVoyager. Four experimental runs were included in this analysis for each participant (one experimental run was excluded for use as a functional localizer, as described in the following section). Since the randomize plug-in does not support ANOVA testing, comparisons between groups were performed using two-sample unpaired *t*-tests with 1000 permutations, threshold free cluster enhancement (TFCE), and FDR thresholding of *q* < 0.01.

To perform region of interest (ROI) analyses, one of the audiovisual block design imaging runs collected from each participant was used as a functional localizer. Group analyses were performed on these localizer runs using multi-subject GLMs computed separately for each of the three experimental groups (i.e., ME, BV, MV). This approach resulted in a group mean map of activity associated with each experimental contrast of interest for each group of participants. Maps to localize the auditory cortex were produced by using activity associated with the unimodal auditory stimuli condition relative to baseline. The visual cortex was localized using activity associated with the unimodal visual stimuli condition relative to baseline. “Audiovisual” regions were isolated by contrasting activity in the synchronous audiovisual condition versus activity in the auditory and visual conditions (i.e., audiovisual > auditory + visual). These group maps were imported into Neuroelf v1.1^1^ where conjunction maps were computed for (1) the BV and ME participant groups, (2) the MV and ME groups, and (3) the BV and MV groups to produce ROIs associated with each of the contrasts described above. All voxels included in ROIs had a *p*-value equal to or lower than 0.05 in included groups of participants. This approach was used to help mitigate the possibility of biasing ROI masks toward any one participant group (i.e., to prevent the inclusion of voxels that were only active in one participant group). These ROI masks were then imported back into BrainVoyager where they were applied to analyses of the remaining four functional imaging runs from each participant. Group ROI GLM analyses were performed using these imaging runs, and ANOVAs were used to test for group-related differences in mean beta weight values associated with each region.

#### Experiment 2: McGurk Region of Interest Analysis

**FIGURE 2:**
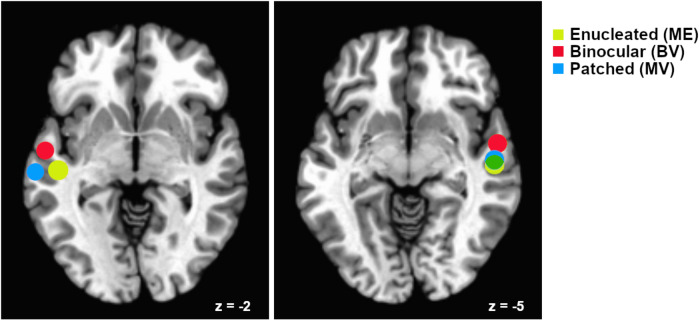
Relative locations of superior temporal gyrus “audiovisual” regions of interest for each participant group. Regions are plotted as spheres (radius = 6 mm) centerd on the mean Talairach coordinates for each group. Left hemisphere regions are shown on an axial slice at Talairach *z* = –2 and right hemisphere regions are shown on an axial slice at *z* = –5. Mean coordinates for the Monocular Enucleation Group (ME) are shown in yellow, Binocular Viewing Controls (BV) in red, and eye-patched Monocular Viewing Controls (MV) in blue. Overlap of the right hemisphere ME and MV regions is shown in green.

Data associated with Experiment 2 were analyzed using the Analysis of Functional Neuroimages (AFNI) software package (Cox, 1996). All five audiovisual block design functional runs were used to create region of interest masks for ROI analyses of the McGurk functional imaging runs. Preprocessing of the audiovisual block design runs included slice time correction, coregistration of functional and anatomical images, transformation of images from subject space to Talairach space, motion correction using coregistration of each functional volume to the volume with the minimum outlier fraction, masking of functional data, within mask spatial smoothing using a 4 mm full width half maximum Gaussian kernel, and scaling of intensity values to a mean value of 100. Pairs of volumes where the Euclidean Norm of the motion derivative exceeded 0.3 mm were removed (in practice, this resulted in very few volumes being removed), and motion estimate parameters were included as regressors of no interest to account for motion-correlated variance in the data. In addition, volumes containing greater than 10% outlier voxel fraction were removed (again resulting in few volumes being removed in practice). Stimulation timing for each condition was provided for GLM analyses as described in Experiment 1. Masks of left and right hemisphere superior temporal regions associated with audiovisual processing were created using a conjunction analysis to isolate voxels equally active (*p* < 0.05) in both the Auditory stimuli and Visual stimuli conditions for each individual study participant. These ROIs were applied in analyses of the McGurk stimuli functional runs (see Figure 2).

Preprocessing of the McGurk runs was performed as described above for the audiovisual block design runs. For these data, a predictor was defined for each condition (i.e., auditory only, visual only, synchronous audiovisual, and McGurk stimuli) and the timing of each stimulus was used in deconvolution of the rapid event-related structure of these imaging runs. Tent functions for deconvolution analysis were centered at TR times. For each participant, ROI masks (localized using the audiovisual block design runs) were applied to the preprocessed McGurk runs. For each condition, mean beta weights associated with each ROI were extracted for each participant. SPSS was used to perform ANOVA comparisons of mean beta weights between participant groups.

## Results

None of the whole brain or ROI analyses performed resulted in any statistically significant differences between any of the participant groups tested. Due to the rare patient group involved in this study and the subsequent small sample size, only descriptive statistics are reported. The data reported are the median and interquartile range (IR) mean beta weight signal in each region. The data reports only participants with an active ROI. See Table 1 (Experiment 1) and Table 2 (Experiment 2) for each group including mean cluster size and TAL coordinates.

**TABLE 1:** Conjunction ROIs containing voxels significantly activated in both participant groups with mean cluster size (SD) and mean Talairach coordinates (STG = superior temporal gyrus, IFG = inferior frontal gyrus).

**Groups**	**Functional ROI**	**Brain region**	**Mean cluster size (mm^3^)**	**Mean (SD) talairach coordinates**		
				**X**	**Y**	**Z**
BV and ME	Low-level auditory	Right STG	837	56.21 (4.53)	−27.69 (3.27)	8.66 (2.06)
	High-level auditory 1	Right STG	3544	53.27 (5.72)	−21.38 (7.14)	4.06 (3.77)
	High-level auditory 2	Left STG	2038	−55.66 (5.97)	−18.23 (5.43)	3.87 (2.52)
	High-level audiovisual 1	Right STG	6096	55.58 (6.07)	−18.20 (8.98)	4.70 (3.81)
	High-level audiovisual 2	Left STG	2084	−52.74 (6.25)	−16.96 (7.31)	3.95 (2.38)
MV and ME	Low-level audiovisual 1	Right Insula	1398	37.89 (5.06)	19.03 (3.47)	10.34 (2.38)
	Low-level audiovisual 2	Right STG	588	55.45 (2.89)	−27.21 (3.15)	6.67 (2.36)
	High-level auditory 1	Right STG	591	49.66 (3.84)	−16.41 (3.47)	6.20 (1.89)
	High-level audiovisual 1	Right STG	2705	54.68 (5.76)	−12.07 (7.85)	3.53 (3.39)
	High-level audiovisual 2	Left STG	1221	−51.67 (5.18)	−16.82 (7.57)	4.28 (2.46)
BV and MV	Low-level auditory 1	Right Precentral Gyrus	1774	42.07 (3.71)	4.87 (3.51)	33.51 (4.80)
	Low-level auditory 2	Right STG	4061	56.06 (5.69)	−18.91 (7.90)	5.71 (3.99)
	Low-level auditory 3	Left IFG	3010	−59.88 (4.67)	−26.39 (7.98)	6.94 (3.38)
	High-level auditory 1	Right STG	4629	56.85 (6.22)	−13.33 (7.58)	3.29 (3.79)
	High-level auditory 2	Left STG	2408	−55.33 (7.32)	−17.22 (7.42)	4.81 (2.27)
	High-level audiovisual 1	Right STG	5200	56.34 (6.24)	−14.74 (6.86)	2.80 (3.99)
	High-level audiovisual 2	Left STG	4683	−55.83 (6.75)	−17.40 (9.26)	3.09 (3.20)

**TABLE 2:** Total number of participants with active ROI, mean (SD) cluster size (mm^3^) and mean (SD) Talairach coordinates for experiment 2: rapid event related design.

**Group (number with active ROI)**	**Functional ROI**	**Mean (SD) cluster size (mm^3^)**	**Mean (SD) Talairach coordinates**		
	
**Audiovisual**			**X**	**Y**	**Z**
BV (*n* = 8)	Left	287.22 (255.99)	−53.50 (4.50)	5.50 (15.52)	−7.50 (6.87)
(*n = 9)*	Right	234.88 (146.76)	54.00 (6.99)	9.00 (14.96)	−5.75 (5.50)
MV (*n* = 8)	Left	143.00 (85.20)	−51.50 (7.19)	14.83 (11.14)	−6.50 (6.54)
(*n* = 9)	Right	78.00 (72.63)	60.00 (7.35)	21.75 (10.96)	2.50 (6.80)
ME (*n* = 6)	Left	88.50 (89.03)	−52.00 (3.99)	17.50 (7.75)	−3.50 (2.68)
(*n* = 6)	Right	81.50 (53.74)	46.50 (6.84)	21.50 (8.20)	0.50 (4.52)

### Experiment 1: Whole Brain Analysis

**FIGURE 3:**
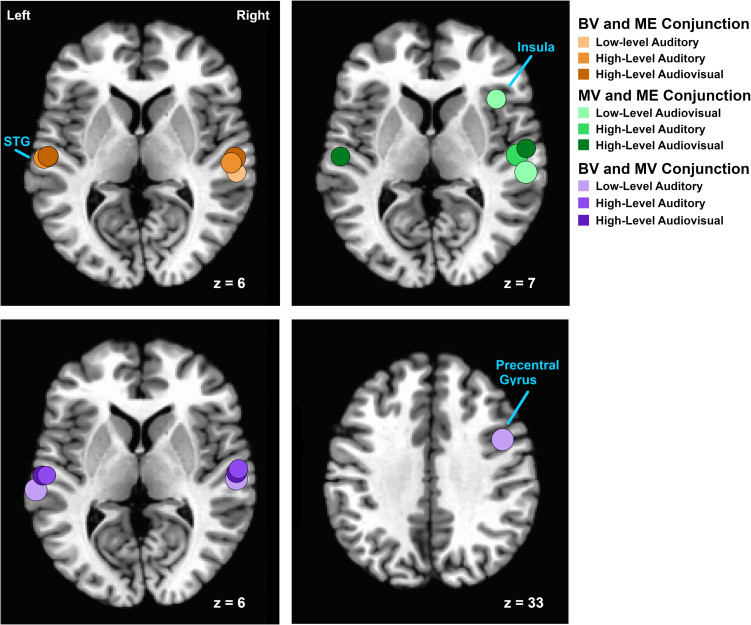
Relative locations of areas isolated using conjunction analyses of functional activation for auditory, visual and audiovisual stimuli between groups. Regions are plotted as spheres (radius = 6 mm) centerd on the mean Talairach coordinates for each pair of groups. Overlapping areas of functional activation for the conjunction analysis between BV and ME groups are shown on an axial slice at Talairach *z* = 6 in shades of orange. Overlapping areas of functional activation for the conjunction analysis between MV and ME groups are shown on an axial slice at Talairach *z* = 7 in shades of green. Overlapping areas of functional activation for the conjunction analysis between BV and MV groups are shown on an axial slice at Talairach *z* = 6 and *z* = 33) in shades of purple.

A conjunction analysis on functional localizer runs was conducted to isolate overlapping areas of functional activation for auditory, visual and audiovisual stimuli between groups. The overlapping ROIs localized for each group comparison are depicted in Figure 3.

**FIGURE 4:**
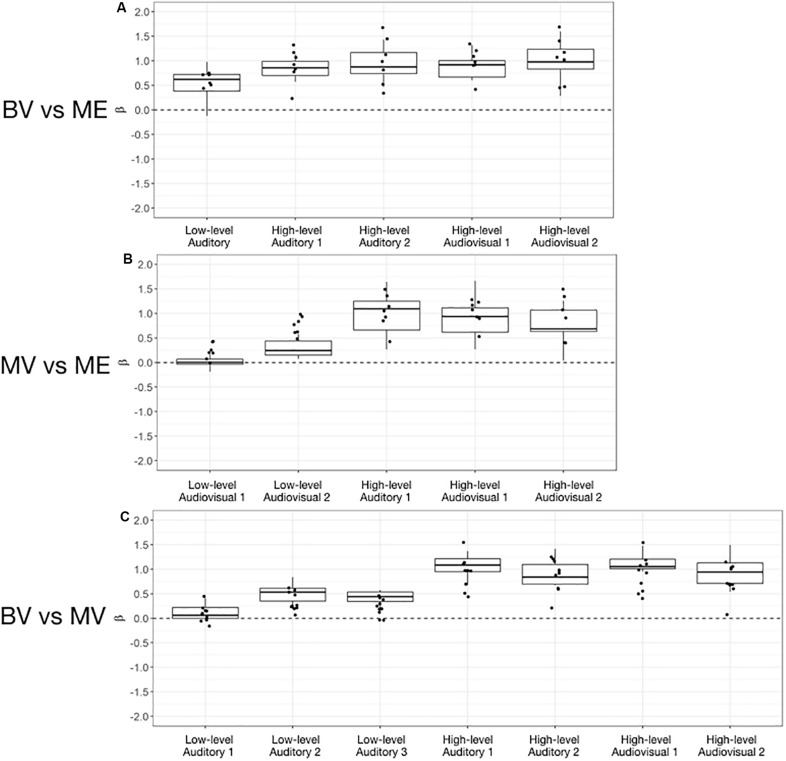
Box and whisker plots of the distribution of peak beta weights for the control groups for the identified auditory, visual and audiovisual ROIs. The horizontal line within each box represents the median of the BV group **(A,C)** and the MV group **(B)**. The boxes correspond to the 25th and 75th percentiles, the whiskers correspond to the 5th and 95th percentiles. Individual data points for the ME group are plotted **(A,B)** and MV group **(C)**.

For the BV group (*n* = 10) compared to the ME group (*n* = 7) five ROIs were identified with significant activation in common between groups. These ROIs were applied to the remaining four experimental runs that were not used for ROI localization. People with one eye did not differ in intensity of cortical activation in these common regions of interest compared to binocular viewing controls (see Table 3). Figure 4A shows that the majority of people with one eye were within the 95% confidence interval (CI) of the binocular viewing control group for all identified ROIs.

**TABLE 3:** Median and interquartile range of the mean beta weight signal in each overlapping area of functional activation for auditory, visual and audiovisual stimuli isolated using conjunction analyses in localizer runs.

**Comparison**	**Functional ROI**	**Median (interquartile range)**	
		**BV**	**ME**
BV and ME	Low-level auditory	0.62 (0.38–0.72)	0.72 (0.53–0.72)
	High-level auditory 1	0.86 (0.70–0.99)	0.92 (0.80–1.12)
	High-level auditory 2	0.87 (0.74–1.16)	0.99 (0.66–1.28)
	High-level audiovisual 1	0.92 (0.67–1.00)	0.98 (0.92–1.15)
	High-level audiovisual 2	0.98 (0.83–1.24)	1.07 (0.75–1.29)
		MV	ME
MV and ME	Low-level audiovisual 1	0.003 (−0.04–0.07)	0.20 (0.13–0.33)
	Low-level audiovisual 2	0.25 (0.15–0.44)	0.77 (0.61–0.88)
	High-level auditory 1	1.09 (0.66–1.25)	1.05 (0.89–1.25)
	High-level audiovisual 1	0.94 (0.62–1.11)	1.07 (0.91–1.20)
	High-level audiovisual 2	0.69 (0.63–0.88)	0.91 (0.66–1.21)
		BV	MV
BV and MV	Low-level auditory 1	0.06 (−0.004–0.22)	0.09 (−0.02–0.15)
	Low-level auditory 2	0.53 (0.35–0.62)	0.26 (0.22–0.52)
	Low-level auditory 3	0.44 (0.34–0.54)	0.22 (0.14–0.36)
	High-level auditory 1	1.08 (0.95–1.21)	0.97 (0.76–1.07)
	High-level auditory 2	0.84 (0.70–1.10)	0.94 (0.68–1.11)
	High-level audiovisual 1	1.06 (1.01–1.21)	0.96 (0.59–1.10)
	High-level audiovisual 2	0.94 (0.71–1.13)	0.70 (0.68–1.02)

For the MV group (*n* = 10) compared to the ME group (*n* = 7) five ROIs were identified with significant activation in common between groups. People with one eye had increased intensity of activation for low-level audiovisual stimuli compared to patched viewing controls (see Table 3). Figure 4B shows that 5/7 and 6/7 ME participants were above the 95% CI of the MV group for the low-level audiovisual ROIs 1 and 2, respectively.

For the BV group (*n* = 10) compared to the MV group (*n* = 10) seven ROIs were identified with significant activation in common between groups. Patched viewing controls did not differ in the intensity of cortical activation in these common regions of interest compared to binocular viewing controls (see Table 3). Figure 4C shows that 5/10 MV participants were below the 95% CI of the BV group for the high-level audiovisual ROI.

### Experiment 2: McGurk Region of Interest Analysis

A region of interest analysis was conducted for individual functionally localized audiovisual regions in the left and right hemisphere. Intensity of activation was compared between groups for auditory, visual, audiovisual and McGurk illusory stimuli. Median and interquartile range (IR) mean beta weight signal in each region is listed for each stimulus type in Table 4.

**TABLE 4:** Median and interquartile range of the mean beta weight signal in each ROI for experiment 2: region of interest analysis.

**Group (number with active ROI)**	**Functional ROI**	**Mean (Interquartile Range)**			
		**Auditory**	**Visual**	**Audiovisual**	**McGurk**
**BV**					
(*n* = 8)	Left	0.19 (−0.03–0.39)	0.19 (0.06–0.28)	0.24 (0.11–0.31)	0.007 (−0.05–0.07)
(*n = 9)*	Right	0.16 (0.03–0.29)	0.16 (−0.008–0.22)	0.20 (0.13–0.32)	0.09 (−0.13–0.16)
MV					
(*n* = 8)	Left	0.10 (−0.02–0.23)	0.15 (0.11–0.22)	0.14 (0.06–0.34)	0.13 (0.04–0.31)
(*n* = 9)	Right	0.05 (0.18–0.30)	0.32 (0.03–0.49)	0.15 (0.03–0.36)	0.11 (−0.10–0.31)
**ME**					
(*n* = 6)	Left	0.27 (0.18–0.43)	0.14 (−0.0004–0.37)	0.21 (0.12–0.38)	0.20 (0.11–0.47)
(*n* = 6)	Right	0.28 (0.19–0.38)	0.09 (0.009–0.28)	0.23 (0.14–0.26)	0.18 (−0.08–0.36)

**FIGURE 5:**
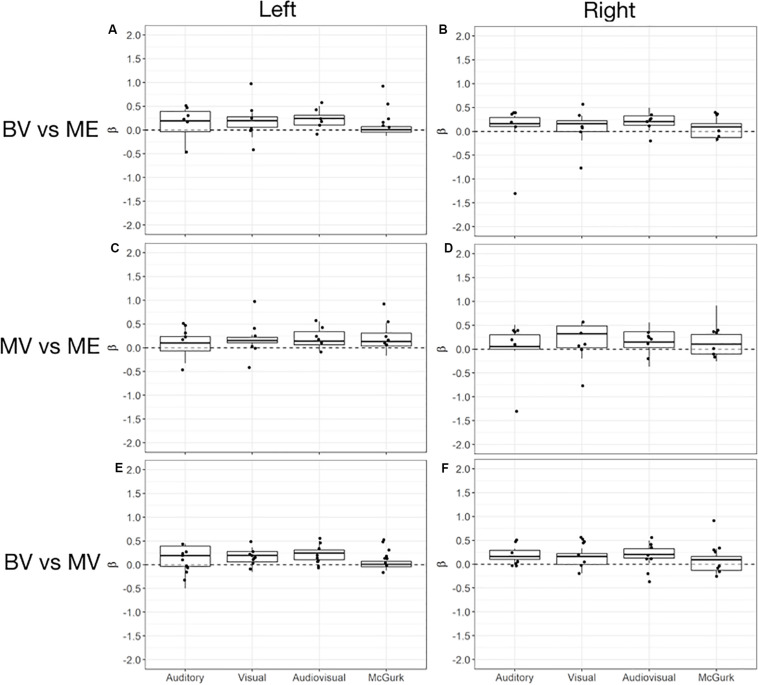
Box and whisker plots of the distribution of peak beta weights for the auditory, visual, audiovisual and McGurk stimulus conditions for the left audiovisual ROI [BV group = **(A,E)**; MV group = **(C)**] and right audiovisual ROI [BV group = **(B,F)**; MV group = **(D)**]. The horizontal line within each box represents the median. The boxes correspond to the 25th and 75th percentiles, the whiskers correspond to the 5th and 95th percentiles. Individual data points for ME group are plotted **(A–D)** and MV group **(E,F)**.

Left and right audiovisual ROIs were identified for the ME (left, *n* = 6; right, *n* = 6) group, BV (left, *n* = 9; right, *n* = 8) group and MV (left, *n* = 9; right, *n* = 8) group. People with one eye have increased intensity of activation in the left audiovisual ROI for McGurk stimuli compared to binocular viewing controls. Patched viewing controls did not differ in intensity of activation compared to people with one eye. Patched viewing controls have increased intensity of activation in the left audiovisual ROI for McGurk stimuli compared to binocular viewing controls. Figure 5 (A, B) show that 5/6 people with one eye were outside the 95% confidence interval (CI) of the binocular viewing control group for the McGurk stimuli for the left audiovisual ROI. Panels (C, D) indicate that the majority of people with one eye were within the 95% confidence interval (CI) of the patched viewing control group. Panels (D, E) indicate that the majority of patched viewing controls were within the 95% confidence interval (CI) of the binocular viewing control group.

### McGurk Effect Behavioral Analysis

**FIGURE 6:**
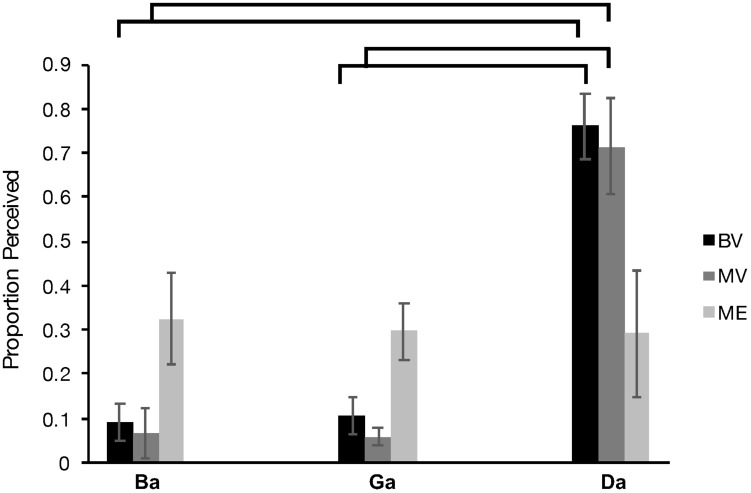
Behavioral McGurk effect perceived (number of times a participant perceived “da” during McGurk trials) for each of the BV (black), MV (gray) and ME group (light gray).

Behavioral performance recorded during the scan session was analyzed. Two of the original 10 MV participants were removed from this data analysis due to performance under chance. A Greenhouse-Geisser corrected (*X*^2^(2) = 13.383, *p* = 0.001), 3 × 3 repeated measures analysis of variance (ANOVA) comparing group (ME vs BV vs MV) and McGurk condition (“Ba”, “Ga”, “Da”) revealed a significant interaction, *F*(2.719, 29.906) = 6.624, *p* = 0.002, η_p_^2^ = 0.376 and main effect of McGurk condition, *F*(1.359, 29.906) = 26.313, *p* < 0.000, η_p_^2^ = 0.545. There was no significant main effect of participant group, *F*(2, 22) = 1.043, *p* = 0.369, η_p_^2^ = 0.087. The BV group demonstrated increased perception of the illusory “Da” condition compared “Ba” (*p* < 0.000) and “Ga” (*p* < 0.000). The MV group demonstrated increased perception of the illusory “Da” condition compared “Ba” (*p* = 0.000) and “Ga” (*p* < 0.000). Furthermore, the ME group did not demonstrate an increase in perception of the “Da” condition compared to the “Ba” condition (*p* < 1.000) and the “Ga” condition (*p* < 1.000). Overall these results indicate a replication of the findings of our previous behavioral study (Moro and Steeves, 2018a). Figure 6 plots the behavioral data for each participant group.

### Behavioral Performance and Functional Activation Correlations

**FIGURE 7:**
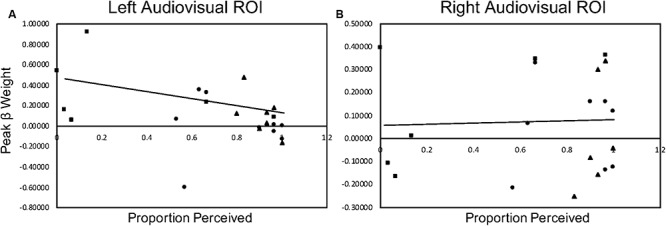
McGurk behavioral performance during scan sessions correlated with peak beta weight activation in the left (column **(A)**) and right (column **(B)**) audiovisual ROI during McGurk trials. The BV group is represented with a circle marker, the MV group is represented with a triangle marker and the ME group is represented with a square marker.

We investigated the relationship between the current behavioral McGurk data obtained during the fMRI session for the BV, MV, and ME groups and the current peak beta weight data for left and right audiovisual ROI’s with McGurk stimuli. Since both of the control groups (BV and MV) demonstrated a McGurk effect and the patient group (ME) did not, we decided to collapse the data and conduct an omnibus correlation in order to accommodate for the small sample size. Non-parametric Spearman correlations indicate a significant correlation, *r*_s_(24) = −0.620, *p* = 0.002 for left audiovisual activation compared to behavioral performance and non-significant correlation, *r*_s_(22) = −0.114, *p* = 0.615 for right audiovisual activation to behavioral performance. Figure 7 plots the behavioral and peak beta weight correlations for the left and right audiovisual ROI.

## Discussion

The current study investigated whether people who had one eye surgically removed early in life have altered functional activation for auditory, visual and audiovisual stimuli. In Experiment 1, a region of interest analysis using ROIs localized with group conjunction analyses was conducted to compare overlapping areas of functional activation for auditory, visual and audiovisual stimuli. When comparing people with one eye to binocular viewing controls five common regions of interest were identified. People with one eye did not differ in intensity of cortical activation in these common regions of interest compared to binocular viewing controls. When comparing people with one eye to patched viewing controls five common regions of interest were identified. People with one eye had a trend for increased intensity of activation When comparing patched viewing controls to binocular viewing controls seven common regions of interest were identified. Patched viewing controls did not differ in the intensity of cortical activation in these common regions of interest compared to binocular viewing controls.

In Experiment 2, a region of interest analysis was conducted for individual functionally localized audiovisual regions in the left and right hemisphere. Intensity of activation was compared between groups for auditory, visual, audiovisual and McGurk illusory stimuli. Both people with one eye and patched viewing controls have a trend for increased intensity of activation in the left audiovisual ROI for McGurk stimuli compared to binocular viewing controls. Patched viewing controls did not differ in intensity of activation compared to people with one eye. Additionally, behavioral performance recorded during the scan session indicates a replication of previous findings (Moro and Steeves, 2018a) where people with one eye do not perceive the McGurk Effect. Correlating behavioral performance on the McGurk task with functional activity yielded a significant negative correlation for the left audiovisual ROI and no significant correlation for the right audiovisual ROI.

The current analyses indicate that in common areas of activation people with one eye and binocular viewing controls do not differ in intensity of activation despite previously observed audiovisual behavioral (Moro and Steeves, 2012, 2013, 2018a,b,c, 2019; Moro et al., 2014) and structural (Kelly et al., 2014, 2015; Moro et al., 2015; Wong et al., 2018, 2019) differences. The lack of difference in activation intensity between these two groups provides evidence that behavioral differences can nonetheless exist in the absence of functional differences.

In contrast, intermediate behavioral performance has been observed for eye patched controls where they fall in between that of people with one eye and binocular viewing controls (Moro et al., 2014; Moro and Steeves, 2018a,b,c, 2019). Consistent with this, the present neuroimaging study indicates functional differences between people with one eye and patched viewing controls. Specifically, people with one eye have a trend toward increased activation intensity in common regions for low-level audiovisual stimuli compared to patched viewing controls. This indicates that short term partial visual deprivation from wearing an eye patch may have a more negative effect on functional activation than long term partial visual deprivation from unilateral eye enucleation. Previous research on monocular deprivation in binocular viewing participants indicates the presence of neuroplasticity in the visual cortex even after short term monocular deprivation through eye patching (see Castaldi et al., 2020 for review). For example, evidence of strengthened cortical excitability after short term monocular deprivation (Lunghi et al., 2015) and enhanced BOLD V1 activation for high spatial frequency stimuli (Binda et al., 2018). These results are restricted to V1, V2, V3, and V4 while not present in V3a and hMT+ (Binda et al., 2018). Results of the current study may indicate that outside of the visual cortex, a decrease in activation intensity may be present. We have speculated that the previously observed intermediate behavioral performance may be the result of binocular inhibitory interactions from wearing an opaque eye patch (Steeves et al., 2004) and may be reflected by reduced functional activation when temporarily wearing an eye patch. Further studies investigating the relationship between behavior and function, within both visual and audiovisual processing regions in long and short term partial visual deprivation should be considered.

A popular tool for studying the mechanisms underlying multisensory integration is the McGurk effect. Susceptibility to the illusion is often inconsistent and shows inter-subject variability possibly due to different cognitive processes that are being used (Beauchamp et al., 2010; Alsius et al., 2017). Its neural substrates have been examined and increased activation of the left STS has been correlated with greater perception of the McGurk effect (Nath and Beauchamp, 2012). Further, clinical populations show differences in their perception of the McGurk effect. People with amblyopia have a reduced susceptibility to the McGurk effect that persists with both binocular and fellow eye viewing (Narinesingh et al., 2014). People with one eye perceive the McGurk effect less often than binocular viewing controls (Moro and Steeves, 2018a). These results have been replicated with the behavioral data obtained during scan sessions in our current study. The present neuroimaging findings indicate people with one eye have a trend toward increased activation intensity for McGurk stimuli along the STS compared to binocular viewing controls. These results are unexpected since behaviorally this group has a much weaker McGurk effect. Since increased activation in the left STS has been shown to be associated with increased perception of the McGurk effect (Nath and Beauchamp, 2012) we expected that the decreased perception of the McGurk effect would be associated with a trend toward decreased activation. The present findings instead show an inverse relationship, as illustrated with a significant negative correlation between behavior and level of brain activity in the left audiovisual ROI. This negative correlation is driven by participants with one eye and indicates that the activation in the left audiovisual ROI for these participants was higher than that reported by the control participants. This finding suggesting perhaps that other cortical regions contribute more heavily to the perception of the McGurk effect in this patient group. It is also possible that the trend toward increased activation may be associated with reorganization of neurons typically dedicated to binocular vision or the remaining eye activating for removed eye, resulting in overall increased activation. These results should be interpreted with caution, however, since the small sample size investigated in this study is not ideal to conduct correlational analyses.

Additional studies examining individual differences that relate brain structure, function and behavioral performance, specifically in sensory deprived individuals should be investigated. As is typical in studying patients with rare diseases, our study was limited due to the rare patient group of people who had one eye surgically removed early in life due to childhood retinoblastoma. It is challenging to obtain a normalized and sufficiently large sample size to conduct inferential statistics and as such to lessen these limitations each patient was sex- and approximately age- matched with participants in both control groups.

In conclusion, the growing body of evidence demonstrates that a number of perceptual accommodations, as well as, structural and functional brain changes occur across the senses in people who have lost one eye early in life. These adaptations likely serve to mitigate the loss of binocularity during early brain development through altered sensory processing compared to binocular and patched viewing controls.

## Data Availability Statement

The datasets generated for this study are available on request to the corresponding author.

## Ethics Statement

The studies involving human participants were reviewed and approved by York University Research Ethics Board. The patients/participants provided their written informed consent to participate in this study.

## Author Contributions

Author contributions by SM include conceptualization, formal analysis, investigation, methodology, visualization, writing (original draft). Author contributions by DG include analysis methodology, visualization, writing. Author contributions by JS include conceptualization, methodology, funding acquisition, project administration, resources, supervision, writing (review and editing).

## Conflict of Interest

The authors declare that the research was conducted in the absence of any commercial or financial relationships that could be construed as a potential conflict of interest.

## Footnotes

1 www.neuroelf.net, RRID:SCR_014147
